# CVE: an R package for interactive variant prioritisation in precision oncology

**DOI:** 10.1186/s12920-017-0261-6

**Published:** 2017-05-25

**Authors:** Andreas Mock, Suzanne Murphy, James Morris, Francesco Marass, Nitzan Rosenfeld, Charlie Massie

**Affiliations:** Cancer Research UK Cambridge Centre, Cancer Research UK Cambridge Institute, University of Cambridge, Cambridge, CB2 0RE UK

**Keywords:** Cancer variant explorer, Melanoma, Prioritization, Personalized oncology, WGCNA, Co-expression network, TCGA, Molecular tumor board

## Abstract

**Background:**

An increasing number of precision oncology programmes are being launched world-wide. To support this development, we present the Cancer Variant Explorer (CVE), an R package with an interactive Shiny web browser interface.

**Results:**

Leveraging Oncotator and the Drug Gene Interaction Database, CVE offers exploration of variants within single or multiple tumour exomes to identify drivers, resistance mechanisms and to assess druggability. We present example applications including the analysis of an individual patient and a cohort-wide study, and provide a first extension of CVE by adding a tumour-specific co-expression network.

**Conclusions:**

The CVE package allows interactive variant prioritisation to expedite the analysis of cancer sequencing studies. Our framework also includes the prioritisation of druggable targets, allows exploratory analysis of tissue specific networks and is extendable for specific applications by virtue of its modular design. We encourage the use of CVE within translational research studies and molecular tumour boards. The CVE package is available via Bioconductor (http://bioconductor.org/packages/CVE/).

**Electronic supplementary material:**

The online version of this article (doi:10.1186/s12920-017-0261-6) contains supplementary material, which is available to authorized users.

## Background

The majority of cancers are believed to be driven by somatically acquired genomic alterations that converge on cancer pathways. The advent of cost-effective, high-throughput sequencing technologies has enabled the systematic cataloging of genomic landscapes of more than 50 tumour entities through national and international projects e.g. *The Cancer Genome Atlas* (TCGA, [1]) and the *International Cancer Genome Consortium* (ICGC, [2]). For every entity, recurrent point mutations, deletions, insertions, translocations and potential new treatment targets were revealed. Pan-cancer analyses have further helped to relate these findings across tumours [3]. In addition, sequencing studies have investigated intratumoural heterogeneity and disease evolution [4, 5]. Meanwhile, analysis of circulating tumour DNA (ctDNA) successfully enabled non-invasive monitoring of the evolution of different tumour clones and treatment resistance over the course of the disease [6–9]. This convergence of discovery, technology and therapeutic development has created an opportunity to test whether systematic knowledge of genomic information can successfully guide targeted therapy and improve patient outcomes (reviewed in [10, 11]).

Owing to the decreasing costs of sequencing for routine diagnostics in clinical oncology [12], an increasing number of cancer centres are switching from sequencing panels of recurrent hotspot mutations to exome sequencing (approaches reviewed in [13]) in search of targetable genetic variants. Here, variant prioritisation remains one of the biggest obstacles because tumour genomes harbour hundreds to hundreds of thousands of somatic mutations [3]. Variant prioritisation in this context describes the process of somatic mutation annotation and subsequent contrasting of evidence to identify ’known driver variants’ and ’likely somatic driver variants’. This includes cancer genes mutated at high frequency, although many more are found to be mutated infrequently. The observation that known cancer driver gene mutations occur at low frequencies in some tumour entities (e.g. only 5% of melanomas harbour hot-spot *IDH1* mutations) suggests that many drivers may yet remain undiscovered due to limited cohort sizes [14]. For variants without prior functional analyses related to cancer, a plethora of computational approaches predicting their functional impact has been developed in recent years. In addition, databases of somatic mutations in cancer are at hand (e.g. COSMIC database [15]).

Precision oncology seeks to leverage this molecular information to improve patient treatment. This is a highly collaborative effort and omics-driven therapeutic decisions are being made in molecular tumour boards consisting of physicians, bioinformaticians and biologists. A number of web applications have been developed to facilitate interactive prioritisation of variants [16–20] (Additional file 1: Table S1). However, to our knowledge, none is purpose-built for precision oncology, enables functional extension (through open-source code) and includes ‘druggability’ information about the prioritised mutated genes.

We developed CVE, the Cancer Variant Explorer, to provide an interactive and flexible application for variant prioritisation to support genomics-driven decision-making. To show the functionality of CVE, we applied it in a single colorectal cancer patient (as a ’molecular tumour board’ example), as well as in a cohort study of 93 *BRAF*-wt/*RAS*-wt melanomas, where druggable targets are poorly understood.

## Implementation

### Overview of CVE workflow

**Fig. 1:**
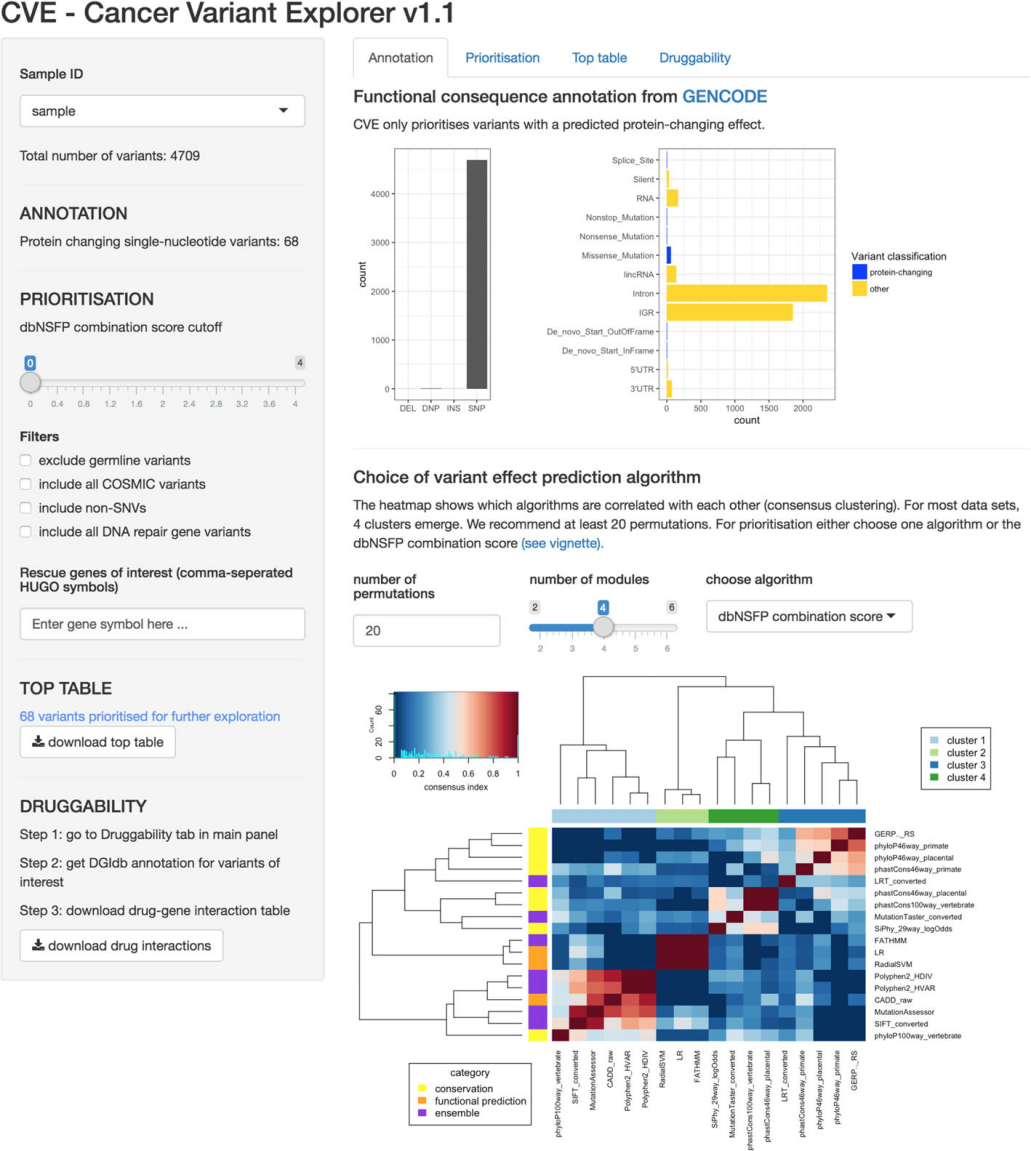
Screenshot of CVE Shiny app. After loading the CVE package, the Shiny app can be started with the function openCVE

**Fig. 2:**
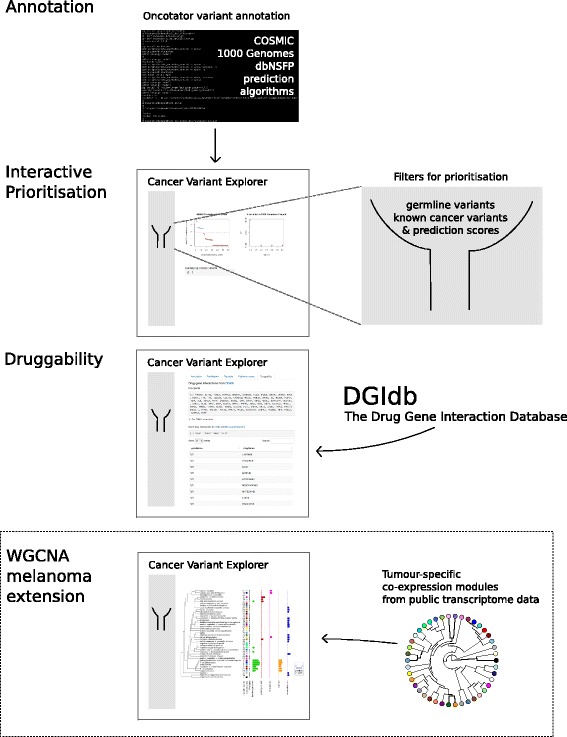
Graphical abstract of CVE workflow. Variants of interest identified in high-throughput sequencing cancer studies are annotated using the Oncotator Variant Annotation tool. Using this annotation, we developed an interactive web application for variant prioritisation named *Cancer Variant Explorer* (CVE). Prioritisation is based on known germline and cancer variants, DNA repair genes and functional prediction scores. Exploration of the tumour-specific pathway context is facilitated using co-expression modules generated from publicly available transcriptome data. Finally druggability of prioritised variants are assessed using the Drug Gene Interaction Database (DGIdb)

Cancer Variant Explorer (CVE) was created using the *Shiny* web application framework for the *R* programming language (shiny.rstudio.com), combining the high functionality of *R* with a concise visualisation of the variant prioritisation process (see Fig. 1 for screenshot of the application interface). We supply the Shiny application CVE in the form of a Bioconductor *R* package to guarantee long-term accessibility and maintenance. The steps of the CVE core workflow to facilitate variant prioritisation are: (i) variant annotation using Oncotator; (ii) exploration of variant annotations; and (iii) assessment of druggability using the Drug Gene Interaction database (DGiDb). A graphical abstract of the CVE workflow is depicted in Fig. 2. In the following implementation sections, the individual steps of CVEs are described in more detail. A step-by-step tutorial on the usage of CVE is also included within the Bioconductor vignette.

### Annotation of variants using Oncotator

The input file for CVE is a comma-separated (csv) file of the format displayed in Additional file 2: Table S2. Before running the interactive CVE application the variants are annotated with the recently released Oncotator Variant Annotation tool summarising variant-centric information from 14 different publicly available resources relevant for cancer researchers [21]. The Oncotator data sources used are summarised in Table 1. The Oncotator annotation can be retrieved using a function provided in the CVE package. By leveraging the Oncotator resource aggregation platform, the annotations used in CVE remain updated and flexible to the incorporation of additional annotations in future.

**Table 1:** Oncotator data sources used in workflow

Annotation category	Resource	Comments
Genomic	GENCODE	Variant classification and mapping to gene
	Human DNA Repair Genes	Curated list from [40]
Protein	UniProt	Protein-specific annotation
	dbNSFP	Conservation and prediction scores
Cancer variant	COSMIC	Catalogue of Somatic Mutations in Cancer
Non-cancer variant	1000 Genomes Project	Germline SNVs

### Exploration of variant annotation

After launching the interactive CVE application (within R, RStudio or by opening a web browser page, if running CVE on a server), the interactive prioritisation begins with the exploration of the Oncotator annotation. The ’functional consequence’ annotation from GENCODE and UniProt classifies the variant into protein changing and non-protein changing as well as into single nucleotide (SNP), double nucleotide polymorphism (DNP) and deletion (DEL). The COSMIC database annotation denotes whether a variant has been found previously in human cancers, and the 1000 Genomes project data denotes whether a variant has been found in germline samples. The workflow aims to identify somatic driver variants, therefore we would have more confidence in protein changing variants that are recurrently mutated in cancer and that are unlikely to be germline variants. In addition information is provided as to whether a variant is in a known DNA repair gene. Variants affecting DNA repair genes are of particular therapeutic importance in cancer (and may predict sensitivity or resistance to a given treatment).

Additional information on the predicted functional impact of each variant is also included, providing lower priority evidence to predict ’possible driver mutations’ in lower frequency variants. This additional information is collated from an increasing list of variant effect prediction algorithms. New algorithms are continuously added to the dbNSFP database, these are also included and updated in the Oncotator annotation.

The prediction algorithms primarily exploit the reasoning that more deleterious gene regions have fewer observed substitutions across species due to tighter evolutionary constrains (i.e. conservation-based algorithms) or the different physico-chemical properties of amino acids and the corresponding three-dimensional protein structure (i.e. functional prediction algorithms). In addition, ensembl scores combining different approaches have been developed (e.g. CADD [22]). For more information about the individual algorithm, see http://portals.broadinstitute.org/oncotator/help/.

Of note, CVE does neither benchmark the scores of the functional prediction algorithms, nor tries to derive the best score. Instead, it displays the heterogeneity of predication based on the rankscores of the 18 algorithms in the current build of the dbNSFP database (Table 2). Rankscores are given a value between 0 and 1, where 1 indicates the highest rank among the 87,347,043 possible non-synonymous single-nucleotide variants in the human genome. CVE depicts algorithms with similar rankscores for a set of variants by means of a heatmap of the consensus indices derived by consensus clustering [23]. The consensus clustering methodology is illustrated in Additional file 3: Figure S1 and the combination score is explained below. However, the user of CVE can choose to use the ranks for one particular algorithm, resembling one cluster of scores, or the combination score.

**Table 2:** Mutation effect prediction algorithms in dbNSFP database. Assignment to a category was made based on their main working principle

	Score name	Category
1	PhastCons100way_vertebrate	conservation
2	PhastCons46way_placental	conservation
3	PhastCons46way_primate	conservation
4	PhyloP100way_vertebrate	conservation
5	PhyloP46way_placental	conservation
6	PhyloP46way_primate	conservation
7	SiPhy_29way_logOdds	conservation
8	GERP++	conservation
9	FATHMM	function prediction
10	LRT	function prediction
11	MutationAssessor	function prediction
12	MutationTaster	function prediction
13	Polyphen2_HDIV	function prediction
14	Polyphen2_HVAR	function prediction
15	SIFT	function prediction
16	LR	ensemble score
17	RadialSVM	ensemble score
18	CADD	ensemble score

We propose that the maximum useful information can be derived from the dbNSFP combination score *c* for the rankscore of algorithm *i* in cluster *j* determined by consensus clustering

1 $${} c = \sum_{j= 1}^{m} y_{j} \qquad \text{with} \qquad y_{j} = \begin{cases} \overline{x_{ij}} & \text{if} \quad \overline{x_{ij}} \geq 0.75\\ 0 & \text{if} \quad \overline{x_{ij}} < 0.75 \end{cases}  $$

where *x*_*ij*_ is the rankscore of algorithm *i* in cluster *j* and $\overline {x_{ij}}$ the mean rankscore of algorithm cluster *j*. $\overline {x_{ij}}$ is only added to *c* if there is significant evidence for the variant in algorithm cluster *j*, defined by a mean rankscore belonging to the upper quartile of rankscores.

### Assessment of druggability

The final step required to guide precision cancer medicine is the assessment of the druggability of candidate variants. The Drug-Gene Interaction database (DGIdb, [24]) offers a comprehensive collection of drug-gene interactions from six sources: PharmGKB [25]; The Therapeutic Target Database (TTD) [26]; the ’targeted agents in lung cancer’ (TALC) publication [27]; the ’trends in the exploitation of novel drug targets’ (TEND) publication [28]; and My Cancer Genome [29]. Within the CVE workflow we retrieve data from TEND and My Cancer Genome, because both sources are expert-curated and comprise data from multiple tumour types. Within the TEND data, we only used antineoplastic agents (for detailed description of drug classes summarized in this group, see supplemental material of [24]). Of note, a gene will be deemed druggable in CVE independent of the tumour entity for which the drug was approved. This is consistent with the increasing trend of administering targeted therapies "off-label", after progression on standard of care treatment. However, it should be made clear that treatment efficacy in a specific molecular subtype cannot be accurately predicted in the absence of clinical trial data. CVE accesses the DGIdb data via the application programming interface (API). This way, a local installation of the database is not required and the entries retrieved include the most up-to-date annotations.

### Cohort case study in melanoma

*BRAF* and *RAS* hotspot mutations occur frequently in cutaneous melanomas and can be targeted with *BRAF* and *MEK* inhibitors, respectively. However, ≈ 27% of tumours do not harbour *BRAF* or *RAS* hotspot mutations and are lacking suitable targeted treatment options. To this end, we gathered a case study from TCGA data with the aim to identify drivers and putatively druggable variants in *BRAF*-wt/*RAS*-wt melanomas. 93 of the 345 patients could be classified as *BRAF*-wt/*RAS*-wt according to the TCGA definition [30]. A ’maf’ file containing the single nucleotide variant (SNV) data of TCGA melanoma patients were downloaded from the TCGA data portal at August 13th, 2015. A list of the TCGA barcodes and the *BRAF*/*RAS* classification are appended in the Additional file 4.

### Generation of melanoma-specific co-expression network

A tutorial describing the generation of a weighted gene co-expression network analysis (WGCNA) from *The Cancer Genome Atlas* (TCGA) RNA-seq data using the WGCNA *R* package developed by Langfelder and Horvarth [31] is included as a vignette in the Bioconductor page of CVE. To construct the melanoma-specific co-expression network, publicly available RNA-sequencing data from 472 melanoma samples were downloaded from the TCGA (The Cancer Genome Atlas) on December 14th, 2015. Curated metadata was obtained for a subset of 332 patients from the most recent TCGA publication [30]. The study set included both primary and metastatic tumour samples, as included in the original TCGA publication. For reproducibility, the TCGA barcodes of the samples can be found in the Additional file 5.

**Fig. 3:**
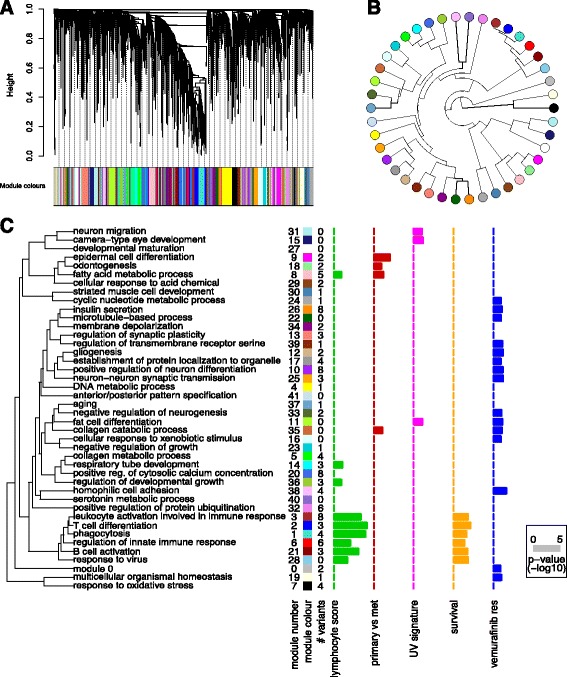
Weighted co-expression network analysis. **a** Gene dendrogram obtained by average linkage hierarchical clustering of TCGA melanoma RNA-seq data (n=472). Colour bar under the plot shows the module assignment determined by the Dynamic Tree Cut algorithm. **b** Eigengene phylogram of the 41 co-expression modules and module 0 (grey dot at 2 o’clock), which contains all genes not included in any of the co-expression modules. **c** Graphical summary of the module significance for the 5 gene significance measures as displayed in *Cancer Variant Explorer*. Modules are named according to the most significant GO term in the enrichment analysis with less than 100 genes per term (to exclude uninformative, high-level GO terms). Barplots show the average absolute gene significance measure per module, i.e. the module significance

To identify co-expression modules in the 472 melanoma patients, genes were next clustered based on the dissimilarity measure, where branches of the dendrogram correspond to modules. The gene dendrogram obtained by average linkage hierarchical clustering is depicted in Fig. 3 a. Ultimately, gene co-expression modules are detected by applying a branch cutting method. Here, we employed the dynamic branch cut method developed by Langfelder and colleagues [32], because constant height cutoffs perform sub-optimally on complex dendrograms. By applying WGCNA in this way to the 472 TCGA melanoma samples we identified 41 co-expression modules. All other genes that were not significantly co-expressed within one of these modules are summarized in module 0 for subsequent analysis (grey colour at 2 ’o’clock in the circular eigengene phylogram; Fig. 3 b). The relationship between the identified co-expression modules can be visualized by a dendrogram of their *eigengenes* (Fig. 3 b). The module *eigengene* is defined as the first principal component of its expression matrix. Module *eigengenes* were highly correlated with the gene that had the highest intramodular connectivity [33]. An advantage of co-expression network analysis is the possibility to integrate external information (such as clinical features). At the lowest hierarchical level, *gene significance* (GS) measures can be defined as the statistical significance (i.e. *p*-value, *p*_*i*_) between the *i*-th node profile (gene) *x*_*i*_ and the sample trait *T*: *GS*_*i*_=−*log* *p*_*i*_.

*Module significance* in turn can be determined as the average absolute gene significance measure. This conceptual framework can be adapted to any research question. For the exploration of variants in a melanoma-specific pathway context, five GS measures were used: (i) lymphocyte score; (ii) survival association; (iii) UV signature; (iv) comparative analysis of primary and metastatic tumours; and (v) vemurafenib resistance. The first 4 GS measures were derived from clinical metadata of the TCGA samples. The samples were dichotomized according to lymphocyte score (histopathological quantification of lymphocyte infiltration in melanomas), the presence of a UV signature as defined by Brash and coworkers [34], or primary vs. metastatic tumours and comparative statistics performed (unpaired two-sided Welch’s t-test). The effect size was the absolute difference in average expression between the groups. For survival analysis, post-accession survival was used as a clinical endpoint in univariate cox proportional hazard models, with the hazard ratio as the effect size. Lastly, to identify variant genes contributing to vemurafenib resistance on the transcriptional level, we derived GS measures using a cell-line dataset recently published by Parmenter and colleagues [35].

To enable a high-level interpretation of the dendrogram of module eigengenes, gene ontology (GO) enrichment analysis was performed for the module genes using the GOstats *R* package [36]. Modules were named according to the most informative GO term with less than 100 genes per term. The *module membership* is defined as *K* ^*q*^=|*cor*(*x*_*i*_,*E* ^*q*^)| where *x*_*i*_ is the profile of gene *i* and *E* ^*q*^ is the eigengene of module *q*. Based on this definition, *K* describes how closely related gene *i* is to module *q*. Figure 3 c illustrates the graphical summary of module significance for five melanoma relevant parameters (GS measures). This plot helps to guide the interpretation of variants on the level of co-expression modules in CVE. A cluster of 9 co-expression modules was significantly associated with the lymphocyte infiltration score. Intriguingly, all 6 survival (post-accession) associated co-expression modules were also significantly associated with the lymphocyte score. In addition, 4 modules were associated with tumour relapse (epidermal cell differentiation, odontogenesis, fatty acid metabolic process, collagen catabolic process). Interestingly, on the transcriptional level, vemurafenib resistance in this cell line derived data [35] is defined by differences in multiple co-expression modules, suggesting either a broad spectrum of changes or a lack of a clear relationship between the cell line study and our tumour tissue-derived co-expression networks.

Together these data provide a framework for the tumour-specific annotation of novel ’putative events’ in melanoma.

## Results

The core implementation of the CVE Shiny app comprises four interactive tabs (i) Annotation, (ii) Prioritisation, (iii) Top table and (iv) Druggability. In the following sections we describe the settings and results for a cohort case study in 93 BRAF-wt/RAS-wt melanomas, as an example implementation for variant exploration using CVE. In addition, we present a first extension of CVE functionality for the exploration of variants in melanoma using a tumour-type specific co-expression network (see Implementation for details).

### Annotation tab

The CVE annotation tab displays a total of 1084 protein-changing single-nucleotide variants (SNVs) from the 93-patient melanoma cohort. The Annotation tab also displays the variant classification (e.g. missense, nonsense, frame-shift etc.) as well as a heatmap of the clusters of prediction algorithms for the dataset. As shown in Additional file 3: Figure S1, four algorithm clusters were identified for the melanoma cohort. Based on the heatmap, the user has the option to either select a single prediction algorithm resembling the information of one algorithm cluster or the proposed dbNSFP combination score (that aims to collapse redundant information). For users unfamiliar with the specific implementations underlying each algorithm, we recommend using the dbNSFP combination score (which was used for prioritisation in our 93-patient melanoma case study).

### Prioritisation tab

The proritisation tab is central to the interactive process of identifying variants of interest. Firstly, common germline variants identified by the 1000 Genomes project can be filtered from further analysis. In the melanoma case study, 67 of the 1084 protein changing SNVs were possible germline variants (a common artifact in many somatic mutation calling pipelines). To exclude all possible germline variants a filter ’check-box’ located on the left side panel can be applied, alternatively a threshold can be set to include variants present at low frequencies in germline data sets (e.g. DNMT3A R882 [37]), depending on the required stringency. Secondly, variants overlapping COSMIC annotations are displayed, to allow the prioritisation of recurrently mutated sites that are ’likely driver events’. In the case study there were 167 overlapping COSMIC variants (i.e. mutated in other cancer samples), a subset of which were frequently altered in cancer. Here, depending on the aim of the analysis all of the 167 overlapping COSMIC variants can be included for further analysis by applying a filter located on the left side panel. Similarly, mutations in DNA damage repair genes can be included in further analysis using a filter located on the left panel of CVE. Finally, the user has the option to interactively choose variant effect prediction algorithms and cutoff thresholds for functional predictions (please see the Implementation section for detailed descriptions of the dbNSFP combination score). This step summarises the predictions that a given variant has a functional impact, using either individual variant effect prediction tools or the combination score that we recommend (see Implementation for details of this consensus clustering). We suggest the use of cutoff thresholds above 1, as this indicates evidence in at least 2 algorithm clusters. For users familiar with the functional prediction algorithms, CVE also offers the functionality to choose individual scores. In the case study, we aimed to obtain a list of ≈ 50-150 prioritised variants suitable for targeted panel sequencing and choose a dbNSFP combination score cutoff of 2. This led to 143 variants in the melanoma case-study. Plots illustrating the number of variants at a chosen cutoff together with annotations for 1000 Genomes, COSMIC and DDR gene variants are displayed within the prioritisation tab at this stage.

### Top table tab

A table of the prioritised variants shortlisted up to this point can be accessed in the next tab of the CVE application. For streamlined data handling, this top table can also be downloaded as a tab-separated file using the download button in the sidebar (for subsequent visualization in spreadsheet software or downstream analysis). The columns of the top table summarize:

- gene: gene symbol
- protein change: location of amino acid change in protein
- type: SNV, dinucleotide substitution (DNP), deletion (DEL) or insertion (INS)
- classification: functional consequence annotation from GENCODE
- score: (rank)score of the mutational effect prediction algorithm selected
- COSMIC entity: number of mutations identified per tumour entity in the COSMIC database

### Druggability tab

Using annotations from the Drug-Gene Interaction database (DGIdb) CVE also allows exploration of potentially druggable targets within a given dataset. To show the full spectrum of evidence for or against a druggable variant in the melanoma case study, no dbNSFP combination score cutoff was initially set. Table 3 summarises all drug-gene interactions available for all 1084 SNVs. However, as previously stated, the user of CVE has full flexibility to adjust these settings at any point of the analysis within the CVE application. The highest confidence variants would be those annotated to be recurrently mutated in the COSMIC database and/or with additional weight for variants with a high dbNSFP score. In contrast, the efficacy of a drug against a mutated gene is less likely where there is little supporting evidence of the mutation having a functional impact (e.g. from COSMIC annotations or dbNSFP scores). Altogether, putatively druggable variants were found in ≈ 25% (23 of 93) of *BRAF*-wt/*RAS*-wt melanoma patients, with 6 patients having two possible drug-gene combinations. The largest drug class identified for this cohort are tyrosine kinase inhibitors that could be potentially used in ≈ 15% of cases (14 of 93).

**Table 3:** Druggability case study. The protein coding change for variants are shown separated by a colon after the gene symbol. Databases listing the drug-gene interaction are abbreviated (T=TEND, M = My Cancer Genome)

Variant	Patient id	dbNSFP score	COSMIC	Drug	Database
EPHA2:p.S790F	26, 52	1.768360		tyrosine kinase inhibitor	T
EPHA2:p.E607K	48, 87	1.737764		tyrosine kinase inhibitor	T
GART:p.S635F	50, 56	0		folate antimetabolite	T
KDR:p.S1100F	16, 45, 75	2.679266		tyrosine kinase inhibitor	T & M
KIT:p.K642E	3, 27, 31, 70	2.454228	yes	tyrosine kinase inhibitor	T & M
KIT:p.V559A	25, 38	2.650527	yes	tyrosine kinase inhibitor	T & M
LHCGR:p.E206K	44, 45	0.77191	yes	GnRH agonist	T
MS4A1:p.G115E	52, 79	0		anti-CD20 antibody	T & M
MTOR:p.A1105T	65, 66	0.8908875	yes	mTOR inhibitor	T & M
PDCD1:p.E211K	34, 51	0		anti-PD1 antibody	M
PIK3C2G:p.E1231K	23, 48	0		PI3K inhibitor	M
PRKCB:p.R361Q	44, 50	0.959385		protein kinase C inhibitor	M
ROS1:p.P1539S	20, 26	0		tyrosine kinase inhibitor	M

### CVE extension: Melanoma co-expression network tab

The four tabs in the core implementation of CVE can be applied to all tumor entities. To illustrate the functionality and flexibility of an open-source *R* package, we developed a first extension to explore the variant genes in a melanoma-specific co-expression network. A vignette describing the generation of the co-expression network using the WGCNA *R* package [31] can be found on the Bioconductor site (and is outlined in the Implementation section).

Figure 3 c summarises the number of variant genes per co-expression module (# variants column) for the following prioritisation settings: (i) dbNSFP combination score cutoff >2; (ii) exclude 1000 Genomes Project variants; and (iii) include all COSMIC variants. Of the resulting 278 variants, 122 occurred in genes that were part of the top 5000 most variant genes of the co-expression network (consistent with a functional role for these genes in melanoma biology). A total of 24 mutated genes fall into the cluster of co-expression modules associated with the lymphocyte infiltration score and overall survival. To further explore an individual module of interest, CVE generates a plot of the module membership over the *p*-value of the respective gene significance measure (e.g. lymphocyte score; Additional file 6: Figure S2). As a third dimension, we weighted the dot-size according to the effect size of a given gene to the module. Additional file 6: Figure S2 depicts the exploration of co-expression module 3 (leukocyte activation involved in immune response), a module that was both significantly associated with the lymphocyte score and post-accession survival. In line with the GO term, all variant genes in this module were involved in leukocyte regulation, with the exception of one multidrug resistance gene (ABCB1). The visualisation of variant genes within the co-expression module enables us to identify variants with a very high module membership, effect size or *p*-value as well as significant associations with the remaining gene significance measures for which a total module significance was not reached. For example, in addition to a significant association with lymphocyte score and post-accession survival, FMO3 is associated with relapse and ADAMDEC1 and ABCB1 with the UV mutation signature (Additional file 6: Figure S2). Hence, this part of the workflow allows us to provide a biological context for variants with likely functional impacts, expediting both further biological and potentially clinical studies.

## Discussion

In recent years, *Precision Cancer Medicine* has developed from a mere buzz word into a framework for clinical decision-making at several comprehensive cancer centres world-wide. On the one hand, cost-effective targeted sequencing approaches are used to assess the ever-increasing number of known cancer drivers often prospectively within clinical trials. On the other hand, genome-wide analyses (i.e. exome sequencing) help to unveil this increasing list, mainly through retrospective comparisons. As more and more data are generated, clinical utility increases, but so does the complexity in data analysis.

A number of web applications have been developed to facilitate interactive prioritisation of variants (Additional file 1: Table S1). BrowseVCF is a comprehensive open source web application based on Python [16], enabling the exploration of VCF files, but requires variant annotation upstream of the tool. Database.bio is another web application for variant prioritisation [17]. However, at the time of writing, neither the tool website nor the supplemental material of the manuscript were available online. The web application Exome Variation Analyzer (EVA) offers multiple modules for variant prioritisation using the commercial IntegraGen annotation [18], but at the time of writing this tool was also not available online. gNOME is a comprehensive web application, including the functionality to explore variant genes within pathways (GO, KEGG). However, no druggability information about the variant gene is available. Lastly, SNVerGUI is a Java application available for multiple platforms, focusing on the exploration of BAM/SAM files, but does not offer a cancer-specific annotation [20]. To our knowledge, no freely available web application is purpose-built for precision oncology and comprises druggability information about prioritised, mutated genes within a single framework. In addition, we believe that in the long run, collaborative efforts using open source programming environments such as *R* will be needed to effectively translate new information for specific diagnostic or research questions.

Arguably the most challenging part of any tool for *Precision Cancer Medicine* is variant prioritisation. Here, variant effect prediction algorithms are an integral part, however the incorporation of additional knowledge databases are crucial to fully support clinical interpretation of cancer genomic data. At this point interactivity and flexibility are key, to allow different criteria to be applied for variant filtering, depending on the scientific question (e.g. druggability in Oncology trials or target discovery studies for drug development). We developed a Shiny web application based on the *R* statistical environment enabling end users (such as biologists and physicians) to interactively apply different cutoffs and filters.

The data sources implemented in this version of CVE include the comprehensive cancer variant annotation tool Oncotator, germline annotations from the 1000 Genomes Project, known cancer-associated variants (COSMIC database), known DNA repair genes and known cancer driver genes. Ultimately, genomics-driven oncology aims at identifying druggable variants, therefore we also include functionality to explore expert-curated sources of drug-gene interactions (TEND and My Cancer Genome, using the DGiDb). Due to the simple programming framework of the application, additional filters and datasets can be implemented in a very short time. This flexibility in adding parameters or data sources to the prioritisation workflow is very valuable and not offered by commercial tools for variant prioritisation (e.g. Ingenuity Variant Analysis). To ensure reproducibility, a spread-sheet with the prioritised variants can be downloaded from the side panel of CVE (including a list of the filters and thresholds applied).

The aforementioned framework for variant prioritisation has the advantage to work independently of the tumour entity. However, this variant-centric annotation focuses on already known cancer drivers and variants, limiting the potential to identify less well characterised variants. A variant prioritisation workflow in the context of clinical trials or translational research should also highlight potentially functional variants that have not been extensively characterized before. In addition, some mutated genes are druggable targets in one tumour entity but not in other tumour types (e.g. vemurafenib is ineffective in *BRAF* V600 mutant colorectal cancers [38]). Therefore, we aimed to explore variants in the tumour-specific pathway context using transcriptomic data. To our knowledge, the current work is the first to employ weighted co-expression network analysis to explore cancer variants in the tumour-specific pathway context. Prerequisite was a large tumour study sample with comprehensive clinico-pathological metadata. Here, all tumour data made publicly available by the TCGA fulfills this requirement. In this work, a melanoma-specific co-expression network was built based on 472 patients. Next, individual variants and co-expression modules were related to sample traits. In this way we could assess associations with (i) the lymphocyte score, (ii) recurrence, (iii) UV signature and (iv) post-accession survival, using the TCGA metadata. In line with the recent transcriptomic characterization of cutaneous melanomas by the TCGA [30], in which the authors describe an immune gene expression subclass associated with an improved patient survival, a cluster of 6 co-expression modules was significantly associated with the lymphocyte infiltration score and in parallel with post-accession survival. In addition, 4 modules were associated with tumour relapse. By exploration of candidate variants in the melanoma-specific co-expression modules, previously unknown variants could be associated with different GS measures. These new findings support the utility of weighted co-expression network analysis for variant prioritisation and provide a starting point for future studies.

The *Cancer Variant Explorer*, is an interactive, iterative and flexible application based on the R programming environment. Using the comprehensive data from the Oncotator Cancer Variant Annotation tool, CVE enables project-tailored variant prioritisation using cancer-relevant databases. Extending current workflows by adding another level of biology it offers the exploration of variant genes in tumour-specific co-expression modules. CVE was applied in a case study of melanoma patients, revealing potentially druggable targets and highlighting genes that have not previously been linked to melanoma and were associated with clinically relevant melanoma gene expression networks.

## Conclusions

Precision Cancer Medicine has great promise to improve the treatment of cancer patients, but several obstacles have to be overcome to increase the success rates of personalised treatments. It will be crucial to more comprehensively identify mechanisms of treatment resistance, especially within the context of the clonal make-up of a tumour. Likewise, robust pharmacogenomic analysis are needed to validate the druggability of candidate variants. In addition, while targeting specific SNVs has been proven successful in some cancers, pan-cancer analysis revealed that not all tumour entities are primarily driven by point mutations, with copy number changes dominating in many cancer types (e.g. ovarian cancer or head and neck squamous cell carcinoma, [39]). Hence, methods for prioritising gene targets within copy number altered regions and integrating these with SNV information may increase the proportion of patients who could benefit from targeted therapies. Ultimately, as genomics-driven oncology is complementary to other therapeutic approaches in oncology, combinations of different treatment approaches are emerging as the next step to improve response rates (such as combinations with immunotherapy, targeting of the tumour microenvironment or cell-based treatments [10]). In the long run, the integration of clinical, pathological and genomic analysis in the context of molecular tumour boards also demands a seamless integration of variant prioritisation tools into hospital IT infrastructures. Given the flexibility of open-source programming structures and web browser implementations, interactive variant prioritisation tools (such as the CVE implementation in Shiny) have the potential to be at the forefront of these developments.

## Availability and requirements

**Project name:** Cancer Variant Explorer (CVE)

**Project home page:** http://bioconductor.org/packages/CVE. CVE will be continuously updated. To work with the latest version, please refer to the development branch in Bioconductor.

**Operating system(s):** Platform independent

**Programming language:** R

**Other requirements:** R 3.3 or higher, R packages: shiny, ConsensusClusterPlus, RColorBrewer, gplots, plyr, ggplot2, jsonlite, ape, WGCNA, RTCGAToolbox

**License:** GNU GPL-3

## Additional files

Additional file 1 **Table S1**. Comparison of tools for interactive variant prioritisation applicable to cancer exomes. (PDF 72 kb)

Additional file 2 **Table S2**. Showing the input variant format of CVE. (PDF 69 kb)

Additional file 3 **Figure S1**. Consensus clustering of dbNSFP rankscores for 1084 protein-changing variants revealed in the case study. Consensus clustering of dbNSFP rankscores for 1084 protein-changing variants identified in the case study. To determine the most meaningful number of clusters of prediction scores, we first assess the consensus CDFs (**Figure S1**A) and the relative change in the area under the CDF curve (*Δ*(*k*), Fig. S1B). Here, *Δ*(*k*) did not increase markedly at more than 5 clusters. Next, using the heatmap of the hierarchical clustering of consensus indexes for the different cluster numbers (**Figure S1**C), we can question the plausibility of clusters in light of the different prediction score categories. This approach revealed that a fifth cluster created another subcluster in the conservation scores only, indicating that 4 clusters could be a more systematic choice. (A) Plot of cumulative distribution functions (CDFs) corresponding to the consensus matrices in the range between 2 and 6 clusters. (B) Relative change in the area under the CDF curve (*Δ*k). (C) Heatmap illustrating the hierarchical clustering of consensus index for 4 clusters of prediction algorithms based on 100 permutations and resampling of 80% of the algorithms and 80% of the variants. Functions provided by the *ConsensusClusterPlus R* package were used to perform the analysis [41]. (PDF 325 kb)

Additional file 4 **Table S3**. Csv table listing the 345 TCGA barcodes of melanoma SNV cohort with classification. (CSV 16 kb)

Additional file 5 **Table S4**. Csv table listing the 472 TCGA barcodes of melanoma RNA-seq cohort used for co-expression network analysis. (CSV 50 kb)

Additional file 6 **Figure S2**. Exploration of prioritised variant genes within co-expression modules from the case study. Exploration of prioritised variant genes within co-expression module 3 (leukocyte activation involved in immune response) for the 5 Gene Significance measures. Module membership is defined as the correlation between the gene profile and the eigengene of module 3. Dots are weighted according to effect size. A *p*-value cutoff of <0.05 is indicated by the vertical dashed line. A short description of the gene function is given. (PDF 20 kb)

## Abbreviations

COSMIC: Catalogue of somatic mutations in cancer
CVE: Cancer variant explorer
DGIdb: Drug gene interaction database
SNV: Single nucleotide variant
TCGA: The Cancer Genome Atlas
WGCNA: Weighted gene co-expression network analysis
wt: Wild-type
