# Supplementary material for: CVE: an R package for interactive variant prioritisation in precision oncology
Source: BMC Med Genomics. 2017 May 25;10:37. doi: 10.1186/s12920-017-0261-6 (PMC5445311; doi:10.1186/s12920-017-0261-6)
Supplement: Supplementary file 1 — Table S1. Comparison of tools for interactive variant prioritisation applicable to cancer exomes. (PDF 72 kb) [file 12920_2017_261_MOESM1_ESM.pdf]

Table S1 Tools for interactive variant prioritisation applicable to cancer exomes. NA: information not available. * supplemental data not accessible on journal website (as of January 2017)

Name	Front-end/back-end	Annotation databases	Druggability info	Cancer-specific	Input data	Open source	Reference
CVE	Shiny application/R	Oncotator	DGiDb	yes	csv table	yes	
Browse VCF	web application/Python	requires prior annotation	requires prior annotation	requires prior annotation	VCF	yes	[15]
database.bio	web application	25 databases*	NA*	NA*	e.g. VCF, BAM	NA*	[16]
EVA	web application	IntegraGen company	no	yes	list of variants	yes	[17]
gNOME	web application	dbNSFP, GO, KEGG	no	no	VCF, GVF	yes	[18]
SNVerGUI	Java application	wANNOVAR	no	no	BAM, SAM	yes	[40]
