# Supplementary material for: CVE: an R package for interactive variant prioritisation in precision oncology
Source: BMC Med Genomics. 2017 May 25;10:37. doi: 10.1186/s12920-017-0261-6 (PMC5445311; doi:10.1186/s12920-017-0261-6)
Supplement: Supplementary file 2 — Table S2. Showing the input variant format of CVE. (PDF 69 kb) [file 12920_2017_261_MOESM2_ESM.pdf]

Table S2 Input variant format of CVE. The input file needs to be provided as a comma-separated (csv) file containing the location information chromosome, start, end, as well as the information about the reference and the observed allele in the displayed order.

chr	start	end	reference allele	observed allele
1	120437782	120437783	GG	TT
1	145273405	145273406	CG	AT
1	153963239	153963239	C	T
