# Supplementary figures and images for: CVE: an R package for interactive variant prioritisation in precision oncology
Source: BMC Med Genomics. 2017 May 25;10:37. doi: 10.1186/s12920-017-0261-6 (PMC5445311; doi:10.1186/s12920-017-0261-6)

A

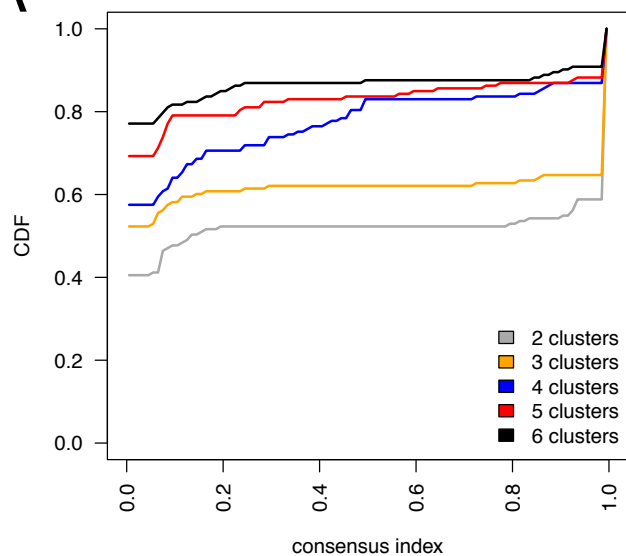

B

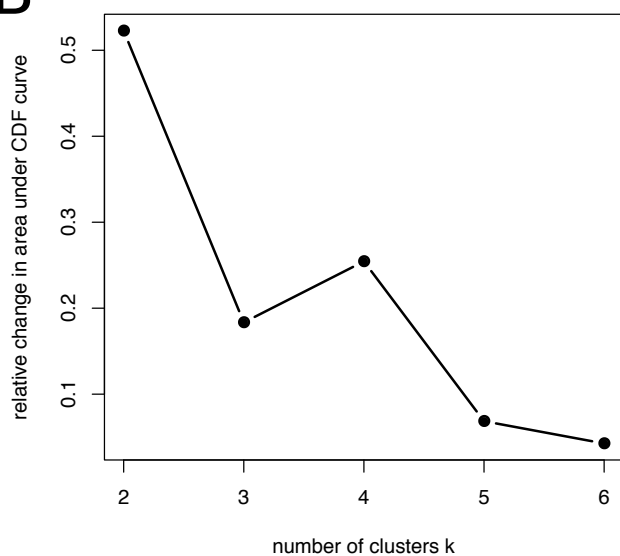

C

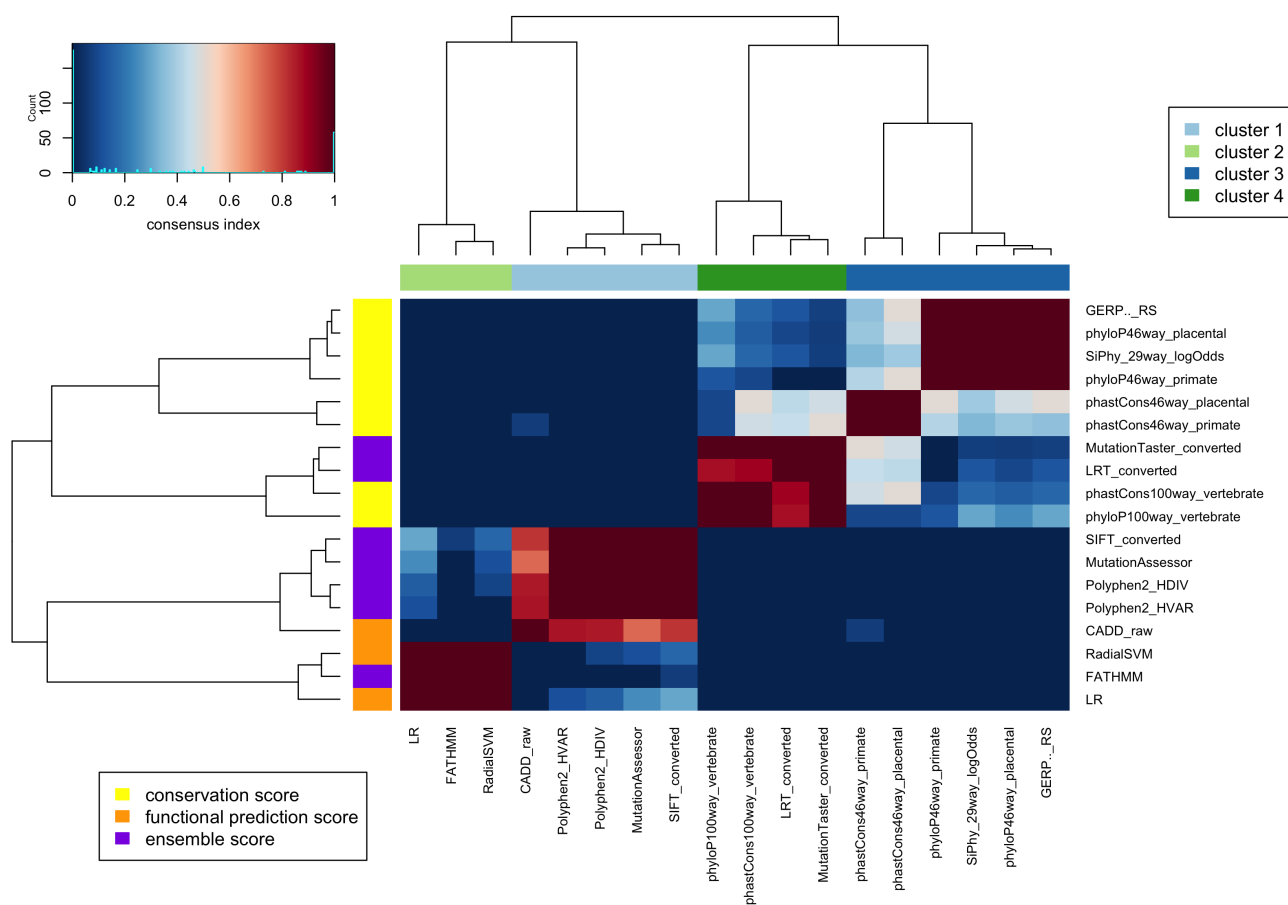

Supplement: Supplementary file 3 — Figure S1. Consensus clustering of dbNSFP rankscores for 1084 protein-changing variants revealed in the case study. Consensus clustering of dbNSFP rankscores for 1084 protein-changing variants identified in the case study. To determine the most meaningful number of clusters of prediction scores, we first assess the consensus CDFs (Figure S1A) and the relative change in the area under the CDF curve (Δ(k), Fig. S1B). Here, Δ(k) did not increase markedly at more than 5 clusters. Next, using the heatmap of the hierarchical clustering of consensus indexes for the different cluster numbers (Figure S1C), we can question the plausibility of clusters in light of the different prediction score categories. This approach revealed that a fifth cluster created another subcluster in the conservation scores only, indicating that 4 clusters could be a more systematic choice. (A) Plot of cumulative distribution functions (CDFs) corresponding to the consensus matrices in the range between 2 and 6 clusters. (B) Relative change in the area under the CDF curve (Δk). (C) Heatmap illustrating the hierarchical clustering of consensus index for 4 clusters of prediction algorithms based on 100 permutations and resampling of 80% of the algorithms and 80% of the variants. Functions provided by the ConsensusClusterPlus R package were used to perform the analysis [41]. (PDF 325 kb) [file 12920_2017_261_MOESM3_ESM.pdf]
