# Supplementary material for: CVE: an R package for interactive variant prioritisation in precision oncology
Source: BMC Med Genomics. 2017 May 25;10:37. doi: 10.1186/s12920-017-0261-6 (PMC5445311; doi:10.1186/s12920-017-0261-6)
Supplement: Supplementary file 6 — Figure S2. Exploration of prioritised variant genes within co-expression modules from the case study. Exploration of prioritised variant genes within co-expression module 3 (leukocyte activation involved in immune response) for the 5 Gene Significance measures. Module membership is defined as the correlation between the gene profile and the eigengene of module 3. Dots are weighted according to effect size. A p-value cutoff of <0.05 is indicated by the vertical dashed line. A short description of the gene function is given. (PDF 20 kb) [file 12920_2017_261_MOESM6_ESM.pdf]

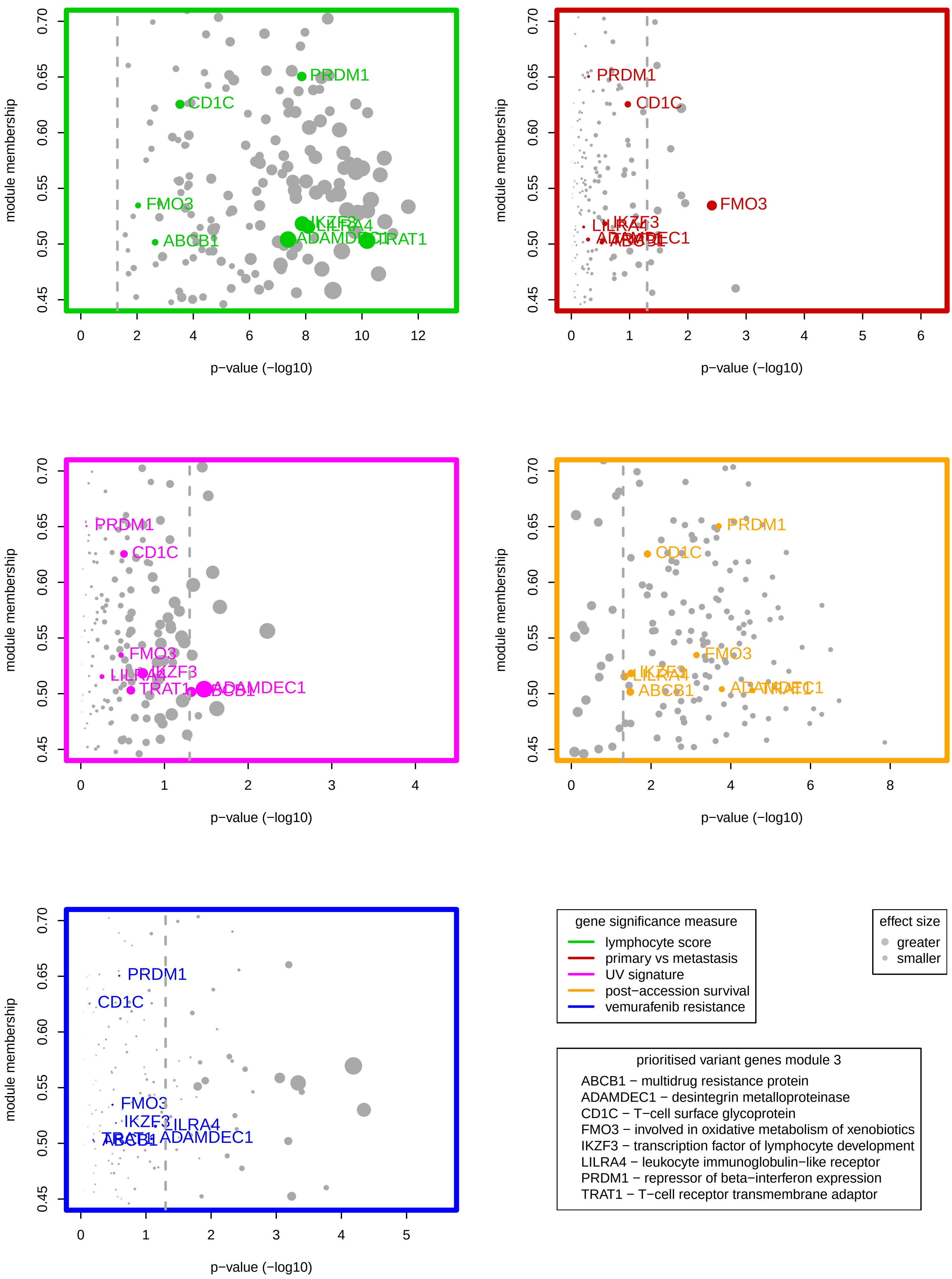
module membership
p−value (−log10)
PRDM1
CD1C
FMO3
ABCB1
IKZF3
LILRA4
ADAMDEC1
TRAT1
gene significance measure
lymphocyte score
primary vs metastasis
UV signature
post−accession survival
vemurafenib resistance
effect size
greater
smaller
prioritised variant genes module 3
ABCB1 − multidrug resistance protein
ADAMDEC1 − desintegrin metalloproteinase
CD1C − T−cell surface glycoprotein
FMO3 − involved in oxidative metabolism of xenobiotics
IKZF3 − transcription factor of lymphocyte development
LILRA4 − leukocyte immunoglobulin−like receptor
PRDM1 − repressor of beta−interferon expression
TRAT1 − T−cell receptor transmembrane adaptor
